# Crystal structure of 9-butyl-6-[2-(pyridin-4-yl)ethen­yl]carbazol-3-amine

**DOI:** 10.1107/S2056989015007975

**Published:** 2015-04-25

**Authors:** Ping Zhang, Xiang-Yang Bai, Ting Zhang

**Affiliations:** a Deparment of Chemistry, Anhui Science and Technolgy University, Fengyang 233100, People’s Republic of China; b College of Chemistry & Chemical Engineering, Anhui University, Hefei 230039, People’s Republic of China, Key Laboratory of Functional Inorganic Materials of Anhui Province, Hefei 230039, People’s Republic of China

**Keywords:** crystal structure, carbazol-3-amine, hydrogen bonding

## Abstract

The asymmetric unit of the title compound, C_23_H_23_N_3_, consists of two mol­ecules, *A* and *B*, with different conformations. In mol­ecule *A*, the dihedral angle between the carbazole ring system (r.m.s. deviation = 0.028 Å) and the pyridine ring is 20.28 (9)° and the N—C—C—C torsion angle of the butyl side chain is −63.4 (3)°. The equivalent data for mol­ecule *B* are 0.065 Å, 48.28 (11)° and 61.0 (3)°, respectively. In the crystal, the components are connected by weak N—H⋯N hydrogen bonds, generating [030] *C*(14) chains of alternating *A* and *B* mol­ecules.

## Related literature   

For background to the applications of carbazoles, see: Wang *et al.* (2013 ▸); Feng *et al.* (2013 ▸); Park *et al.* (2015 ▸). For further synthetic details, see: Zhang *et al.* (2014 ▸).
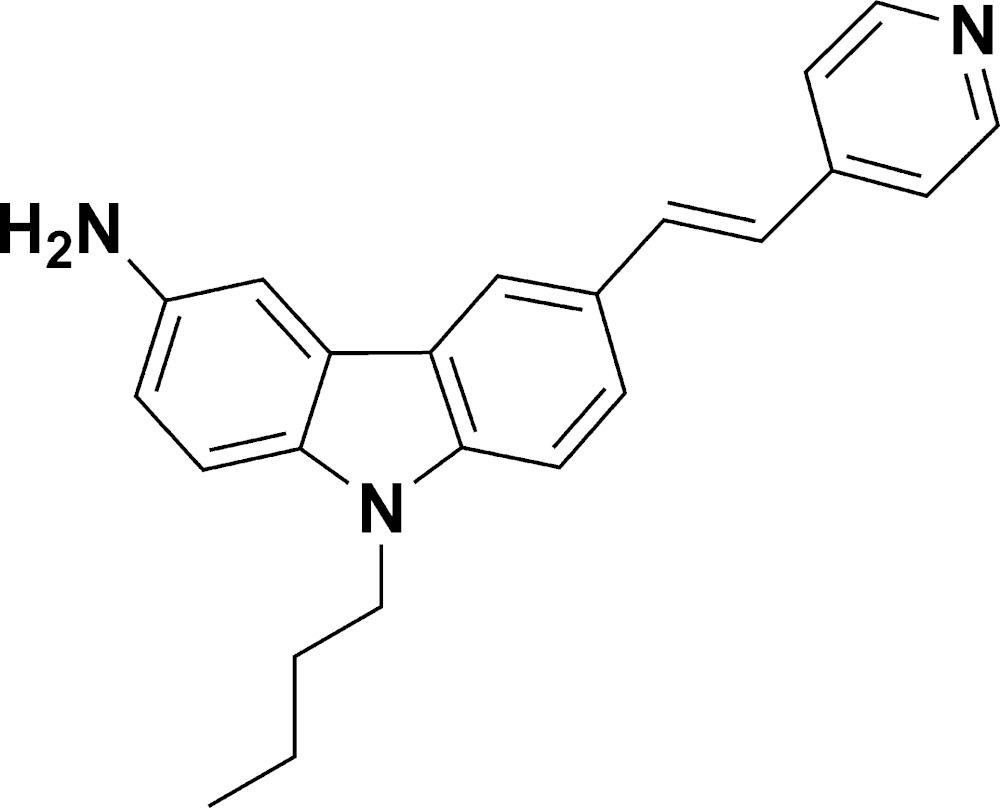


## Experimental   

### Crystal data  

C_23_H_23_N_3_
*M* *_r_* = 341.44
Monoclinic, 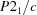

*a* = 11.296 (4) Å
*b* = 18.719 (7) Å
*c* = 17.829 (7) Å
β = 95.362 (5)°
*V* = 3753 (2) Å^3^
*Z* = 8
Mo *K*α radiation
μ = 0.07 mm^−1^
*T* = 296 K
0.30 × 0.20 × 0.20 mm

### Data collection  

Bruker SMART CCD diffractometer
26552 measured reflections
6604 independent reflections
4780 reflections with *I* > 2σ(*I*)
*R*_int_ = 0.029

### Refinement  

*R*[*F* ^2^ > 2σ(*F* ^2^)] = 0.053
*wR*(*F* ^2^) = 0.166
*S* = 1.14
6604 reflections
471 parameters
5 restraints
H-atom parameters constrained
Δρ_max_ = 0.66 e Å^−3^
Δρ_min_ = −0.28 e Å^−3^

### 

Data collection: *SMART* (Bruker, 2007 ▸); cell refinement: *SAINT* (Bruker, 2007 ▸); data reduction: *SAINT*; program(s) used to solve structure: *SHELXS97* (Sheldrick, 2008 ▸); program(s) used to refine structure: *SHELXL97* (Sheldrick, 2008 ▸); molecular graphics: *SHELXTL* (Sheldrick, 2008 ▸); software used to prepare material for publication: *SHELXTL*.

## Supplementary Material

Crystal structure: contains datablock(s) I, Global. DOI: 10.1107/S2056989015007975/hb7392sup1.cif

Structure factors: contains datablock(s) I. DOI: 10.1107/S2056989015007975/hb7392Isup2.hkl

Supporting information file. DOI: 10.1107/S2056989015007975/hb7392Isup3.cml

**Fig. 1.**
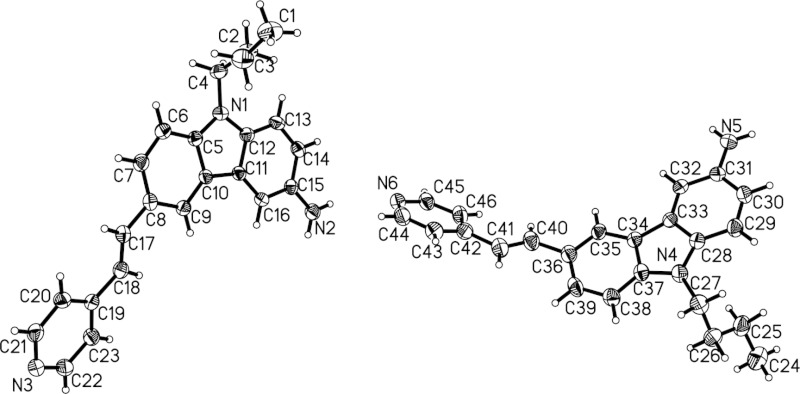
The molecular structure of the title compound.

. DOI: 10.1107/S2056989015007975/hb7392fig1.tif
The mol­ecular structure of the title compound.

CCDC reference: 1061103

Additional supporting information:  crystallographic information; 3D view; checkCIF report

## Figures and Tables

**Table 1:** Hydrogen-bond geometry (, )

*D*H*A*	*D*H	H*A*	*D* *A*	*D*H*A*
N2H2*D*N6	0.86	2.56	3.154(3)	128
N5H5*B*N3^i^	0.86	2.36	3.163(3)	156

Symmetry code: (i) 
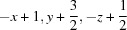
.

## supplementary crystallographic information

### S1. Comment

The title compound is a carbazole derivative with an amine group and a pyridine group. Carbazole is usually utilized in organic functional materials due to it is a large conjugated system with prominent hole-transporting(Wang *et al.*, 2013). The pyridine group havebeen used as heavy metal sensors (Feng *et al.*, 2013) and the amino groupcan be regulated by acid-base based on intermolecular charge transfer(Park *et al.*, 2015). The title compound might be able to get multiple application fields.

In (I) (Fig.1),

The bond distances of C15—N2 is not equal to the bond distance of C31—N5, which are 1.403 Å and 1.395 Å. The torsion angle of C8–C17—C18–C19 are similiar to C36–C40—C41–C42, but the dihedral angles of the phenyl group and pyridine group is different in the two molecular, which are 18.97° and 45.44°, respectively.

The crystal packing shows that the related molecules are linking by N6···H2D—N2, N5···H16—C16 hydrogen bonds.

### S2. Refinement

All hydrogen atoms were placed in geometrically idealized positions and constrained to ride on their parent atoms, with C—H = 0.93 Å and *U*_iso_(H) = 1.2 *U*_eq_.

### Crystal data

**Table:**

C_23_H_23_N_3_	*F*(000) = 1456
*M**_r_* = 341.44	*D*_x_ = 1.209 Mg m^−^^3^
Monoclinic, *P*2_1_/*c*	Mo *K*α radiation, λ = 0.71073 Å
*a* = 11.296 (4) Å	Cell parameters from 7036 reflections
*b* = 18.719 (7) Å	θ = 2.3–24.3°
*c* = 17.829 (7) Å	µ = 0.07 mm^−^^1^
β = 95.362 (5)°	*T* = 296 K
*V* = 3753 (2) Å^3^	0.30 × 0.20 × 0.20 mm
*Z* = 8	

### Data collection

**Table:**

Bruker SMART CCD diffractometer	4780 reflections with *I* > 2σ(*I*)
Radiation source: fine-focus sealed tube	*R*_int_ = 0.029
Graphite monochromator	θ_max_ = 25.0°, θ_min_ = 1.6°
ω scans	*h* = −13→12
26552 measured reflections	*k* = −20→22
6604 independent reflections	*l* = −21→21

### Refinement

**Table:**

Refinement on *F*^2^	Primary atom site location: structure-invariant direct methods
Least-squares matrix: full	Secondary atom site location: difference Fourier map
*R*[*F*^2^ > 2σ(*F*^2^)] = 0.053	Hydrogen site location: inferred from neighbouring sites
*wR*(*F*^2^) = 0.166	H-atom parameters constrained
*S* = 1.14	*w* = 1/[σ^2^(*F*_o_^2^) + (0.0773*P*)^2^ + 0.9436*P*] where *P* = (*F*_o_^2^ + 2*F*_c_^2^)/3
6604 reflections	(Δ/σ)_max_ < 0.001
471 parameters	Δρ_max_ = 0.66 e Å^−^^3^
5 restraints	Δρ_min_ = −0.28 e Å^−^^3^

### Special details

**Table:**

Geometry. All e.s.d.'s (except the e.s.d. in the dihedral angle between two l.s. planes) are estimated using the full covariance matrix. The cell e.s.d.'s are taken into account individually in the estimation of e.s.d.'s in distances, angles and torsion angles; correlations between e.s.d.'s in cell parameters are only used when they are defined by crystal symmetry. An approximate (isotropic) treatment of cell e.s.d.'s is used for estimating e.s.d.'s involving l.s. planes.
Refinement. Refinement of *F*^2^ against ALL reflections. The weighted *R*-factor *wR* and goodness of fit *S* are based on *F*^2^, conventional *R*-factors *R* are based on *F*, with *F* set to zero for negative *F*^2^. The threshold expression of *F*^2^ > σ(*F*^2^) is used only for calculating *R*-factors(gt) *etc*. and is not relevant to the choice of reflections for refinement. *R*-factors based on *F*^2^ are statistically about twice as large as those based on *F*, and *R*- factors based on ALL data will be even larger.

### Fractional atomic coordinates and isotropic or equivalent isotropic displacement parameters (Å^2^)

**Table:**

	*x*	*y*	*z*	*U*_iso_*/*U*_eq_	
C8	0.1402 (2)	0.18126 (11)	0.33640 (12)	0.0506 (5)	
C10	0.07484 (18)	0.28290 (11)	0.26009 (11)	0.0439 (5)	
C11	0.06577 (18)	0.33974 (11)	0.20495 (11)	0.0434 (5)	
C18	0.3040 (2)	0.09517 (12)	0.31670 (12)	0.0526 (6)	
H18	0.3148	0.1176	0.2713	0.063*	
C17	0.2208 (2)	0.12164 (12)	0.35622 (13)	0.0526 (5)	
H17	0.2125	0.0997	0.4022	0.063*	
C12	−0.04171 (18)	0.37514 (11)	0.21275 (11)	0.0457 (5)	
C16	0.14005 (19)	0.36372 (11)	0.15188 (12)	0.0471 (5)	
H16	0.2116	0.3405	0.1465	0.057*	
C15	0.10675 (19)	0.42226 (11)	0.10720 (12)	0.0470 (5)	
C5	−0.02863 (19)	0.28735 (11)	0.29917 (12)	0.0488 (5)	
C9	0.15800 (19)	0.22968 (11)	0.27827 (12)	0.0492 (5)	
H9	0.2252	0.2260	0.2521	0.059*	
C19	0.38121 (18)	0.03355 (11)	0.33784 (12)	0.0464 (5)	
C7	0.0385 (2)	0.18909 (13)	0.37517 (13)	0.0577 (6)	
H7	0.0278	0.1578	0.4145	0.069*	
C13	−0.0760 (2)	0.43368 (12)	0.16804 (13)	0.0526 (5)	
H13	−0.1474	0.4571	0.1732	0.063*	
C20	0.3807 (2)	−0.00335 (13)	0.40535 (13)	0.0559 (6)	
H20	0.3287	0.0102	0.4403	0.067*	
C34	0.5999 (2)	1.02101 (12)	0.11976 (11)	0.0492 (5)	
C33	0.57620 (19)	1.09595 (12)	0.12853 (12)	0.0482 (5)	
C14	−0.0013 (2)	0.45628 (12)	0.11577 (12)	0.0520 (5)	
H14	−0.0236	0.4953	0.0854	0.062*	
C28	0.6780 (2)	1.12522 (12)	0.16791 (12)	0.0525 (5)	
C21	0.4568 (2)	−0.05970 (13)	0.42064 (14)	0.0608 (6)	
H21	0.4538	−0.0833	0.4663	0.073*	
C23	0.4605 (2)	0.00853 (13)	0.28947 (13)	0.0568 (6)	
H23	0.4644	0.0305	0.2430	0.068*	
C37	0.7160 (2)	1.00873 (12)	0.15361 (12)	0.0532 (6)	
C35	0.5315 (2)	0.96430 (12)	0.09044 (13)	0.0554 (6)	
H35	0.4561	0.9729	0.0665	0.066*	
C36	0.5740 (2)	0.89555 (13)	0.09647 (14)	0.0635 (6)	
C6	−0.0456 (2)	0.24026 (13)	0.35797 (13)	0.0582 (6)	
H6	−0.1123	0.2437	0.3846	0.070*	
C31	0.4804 (2)	1.21038 (13)	0.12857 (15)	0.0608 (6)	
C45	0.2326 (3)	0.66774 (13)	−0.00112 (15)	0.0666 (7)	
H45	0.1796	0.6703	−0.0442	0.080*	
C4	−0.2165 (2)	0.36238 (13)	0.28986 (14)	0.0601 (6)	
H4A	−0.2499	0.3222	0.3150	0.072*	
H4B	−0.2673	0.3715	0.2439	0.072*	
C32	0.4770 (2)	1.13893 (13)	0.10920 (13)	0.0557 (6)	
H32	0.4091	1.1196	0.0834	0.067*	
C22	0.5335 (2)	−0.04822 (13)	0.30896 (15)	0.0630 (6)	
H22	0.5850	−0.0634	0.2745	0.076*	
C39	0.6912 (3)	0.88488 (14)	0.12855 (15)	0.0710 (7)	
H39	0.7213	0.8386	0.1311	0.085*	
C26	0.9762 (2)	1.09499 (16)	0.16513 (17)	0.0767 (8)	
H26A	1.0532	1.0988	0.1939	0.092*	
H26B	0.9787	1.0538	0.1324	0.092*	
C42	0.3969 (2)	0.71893 (14)	0.07683 (16)	0.0705 (6)	
C27	0.8826 (2)	1.08206 (15)	0.21935 (15)	0.0713 (7)	
H27A	0.9045	1.0400	0.2493	0.086*	
H27B	0.8819	1.1224	0.2534	0.086*	
C41	0.4903 (3)	0.77281 (15)	0.09790 (17)	0.0784 (8)	
H41	0.5541	0.7593	0.1317	0.094*	
C40	0.4888 (3)	0.83625 (14)	0.07270 (16)	0.0747 (7)	
H40	0.4284	0.8473	0.0354	0.090*	
C3	−0.2190 (2)	0.42683 (15)	0.33998 (15)	0.0681 (7)	
H3A	−0.1855	0.4671	0.3150	0.082*	
H3B	−0.3011	0.4383	0.3465	0.082*	
C38	0.7640 (2)	0.94025 (13)	0.15659 (14)	0.0647 (7)	
H38	0.8418	0.9319	0.1766	0.078*	
C29	0.6807 (2)	1.19679 (13)	0.18829 (14)	0.0644 (6)	
H29	0.7480	1.2164	0.2146	0.077*	
C46	0.3153 (3)	0.72233 (14)	0.01270 (15)	0.0739 (7)	
H46	0.3164	0.7607	−0.0203	0.089*	
C30	0.5828 (2)	1.23771 (14)	0.16907 (15)	0.0659 (7)	
H30	0.5840	1.2855	0.1833	0.079*	
C44	0.3030 (3)	0.61135 (15)	0.10490 (18)	0.0750 (8)	
H44	0.2991	0.5731	0.1378	0.090*	
C43	0.3871 (3)	0.66132 (15)	0.12302 (17)	0.0759 (7)	
H43	0.4381	0.6566	0.1668	0.091*	
C2	−0.1533 (3)	0.41786 (17)	0.41516 (16)	0.0815 (8)	
H2A	−0.0691	0.4136	0.4093	0.098*	
H2B	−0.1787	0.3739	0.4375	0.098*	
C25	0.9563 (3)	1.16126 (16)	0.11666 (18)	0.0816 (8)	
H25A	0.8837	1.1552	0.0837	0.098*	
H25B	0.9451	1.2018	0.1491	0.098*	
C1	−0.1724 (3)	0.48013 (19)	0.46861 (17)	0.0927 (10)	
H1A	−0.1630	0.5245	0.4429	0.139*	
H1B	−0.1150	0.4774	0.5118	0.139*	
H1C	−0.2511	0.4775	0.4846	0.139*	
C24	1.0568 (3)	1.1780 (2)	0.0689 (2)	0.1101 (12)	
H24A	1.1303	1.1805	0.1005	0.165*	
H24B	1.0419	1.2229	0.0439	0.165*	
H24C	1.0620	1.1410	0.0320	0.165*	
N2	0.17911 (18)	0.44634 (10)	0.05257 (10)	0.0597 (5)	
H2C	0.2448	0.4247	0.0466	0.072*	
H2D	0.1578	0.4827	0.0251	0.072*	
N1	−0.09916 (16)	0.34248 (10)	0.26974 (10)	0.0521 (5)	
N4	0.76339 (18)	1.07204 (10)	0.18222 (11)	0.0597 (5)	
N3	0.53538 (18)	−0.08319 (11)	0.37428 (12)	0.0618 (5)	
N6	0.2259 (2)	0.61214 (11)	0.04444 (13)	0.0665 (6)	
N5	0.38357 (19)	1.25534 (13)	0.11005 (17)	0.0895 (8)	
H5A	0.3199	1.2387	0.0860	0.107*	
H5B	0.3875	1.2997	0.1227	0.107*	

### Atomic displacement parameters (Å^2^)

**Table:**

	*U*^11^	*U*^22^	*U*^33^	*U*^12^	*U*^13^	*U*^23^
C8	0.0577 (14)	0.0424 (12)	0.0510 (12)	−0.0030 (10)	0.0014 (10)	0.0007 (10)
C10	0.0447 (12)	0.0384 (11)	0.0484 (11)	−0.0009 (9)	0.0029 (9)	−0.0021 (9)
C11	0.0433 (11)	0.0396 (11)	0.0469 (11)	0.0006 (9)	0.0023 (9)	−0.0047 (9)
C18	0.0575 (14)	0.0531 (13)	0.0472 (12)	−0.0038 (11)	0.0055 (10)	0.0053 (10)
C17	0.0592 (14)	0.0469 (12)	0.0517 (12)	−0.0023 (11)	0.0052 (11)	0.0043 (10)
C12	0.0450 (12)	0.0457 (12)	0.0463 (11)	−0.0003 (10)	0.0034 (9)	−0.0060 (9)
C16	0.0465 (12)	0.0435 (12)	0.0521 (12)	0.0041 (9)	0.0084 (10)	−0.0023 (10)
C15	0.0537 (13)	0.0432 (12)	0.0442 (11)	−0.0033 (10)	0.0039 (10)	−0.0037 (9)
C5	0.0495 (12)	0.0458 (12)	0.0517 (12)	−0.0032 (10)	0.0082 (10)	−0.0044 (10)
C9	0.0482 (12)	0.0468 (12)	0.0529 (12)	−0.0031 (10)	0.0069 (10)	−0.0020 (10)
C19	0.0463 (12)	0.0451 (12)	0.0477 (12)	−0.0054 (9)	0.0040 (9)	0.0005 (9)
C7	0.0666 (15)	0.0529 (14)	0.0554 (13)	−0.0031 (12)	0.0159 (12)	0.0036 (11)
C13	0.0456 (12)	0.0516 (13)	0.0602 (13)	0.0111 (10)	0.0026 (10)	−0.0018 (11)
C20	0.0542 (14)	0.0591 (14)	0.0556 (13)	0.0034 (11)	0.0127 (11)	0.0019 (11)
C34	0.0555 (13)	0.0504 (13)	0.0429 (11)	−0.0032 (10)	0.0107 (10)	0.0069 (10)
C33	0.0518 (13)	0.0491 (13)	0.0451 (11)	−0.0030 (10)	0.0122 (10)	0.0058 (9)
C14	0.0584 (14)	0.0448 (12)	0.0512 (12)	0.0052 (10)	−0.0030 (11)	0.0024 (10)
C28	0.0568 (14)	0.0522 (13)	0.0481 (12)	−0.0015 (11)	0.0024 (10)	0.0037 (10)
C21	0.0619 (15)	0.0604 (15)	0.0609 (14)	−0.0030 (12)	0.0092 (12)	0.0149 (12)
C23	0.0659 (15)	0.0548 (14)	0.0516 (13)	0.0012 (12)	0.0158 (11)	0.0046 (11)
C37	0.0654 (15)	0.0493 (13)	0.0453 (12)	−0.0020 (11)	0.0068 (10)	0.0081 (10)
C35	0.0597 (14)	0.0535 (14)	0.0547 (13)	−0.0080 (11)	0.0152 (11)	0.0005 (11)
C36	0.0759 (16)	0.0565 (15)	0.0606 (14)	−0.0040 (12)	0.0200 (12)	0.0012 (11)
C6	0.0616 (15)	0.0543 (14)	0.0615 (14)	0.0003 (12)	0.0206 (12)	0.0030 (11)
C31	0.0526 (14)	0.0551 (15)	0.0768 (16)	0.0021 (11)	0.0180 (12)	−0.0005 (12)
C45	0.0809 (18)	0.0550 (15)	0.0648 (15)	0.0013 (13)	0.0117 (13)	−0.0078 (13)
C4	0.0469 (13)	0.0658 (15)	0.0693 (15)	0.0013 (11)	0.0150 (11)	−0.0054 (12)
C32	0.0472 (13)	0.0582 (15)	0.0630 (14)	−0.0043 (11)	0.0116 (11)	0.0018 (11)
C22	0.0651 (15)	0.0582 (15)	0.0689 (16)	0.0053 (12)	0.0229 (12)	0.0004 (12)
C39	0.097 (2)	0.0438 (14)	0.0740 (17)	0.0070 (14)	0.0186 (15)	0.0115 (12)
C26	0.0597 (16)	0.081 (2)	0.0854 (19)	−0.0030 (14)	−0.0156 (14)	−0.0105 (16)
C42	0.0752 (16)	0.0636 (15)	0.0761 (15)	−0.0041 (12)	0.0255 (12)	−0.0201 (11)
C27	0.0724 (17)	0.0723 (17)	0.0637 (16)	0.0049 (14)	−0.0221 (14)	−0.0032 (13)
C41	0.0883 (19)	0.0602 (17)	0.0870 (19)	0.0006 (13)	0.0085 (16)	0.0007 (14)
C40	0.100 (2)	0.0579 (16)	0.0699 (17)	−0.0029 (13)	0.0281 (15)	−0.0027 (13)
C3	0.0584 (15)	0.0798 (18)	0.0683 (16)	0.0086 (13)	0.0166 (12)	−0.0035 (13)
C38	0.0719 (16)	0.0569 (15)	0.0645 (15)	0.0077 (13)	0.0023 (13)	0.0149 (12)
C29	0.0673 (16)	0.0580 (15)	0.0661 (15)	−0.0050 (13)	−0.0032 (12)	−0.0074 (12)
C46	0.104 (2)	0.0522 (15)	0.0692 (15)	−0.0013 (15)	0.0285 (13)	0.0020 (13)
C30	0.0719 (17)	0.0514 (14)	0.0755 (17)	−0.0023 (13)	0.0124 (14)	−0.0075 (12)
C44	0.085 (2)	0.0575 (16)	0.083 (2)	0.0022 (15)	0.0112 (17)	0.0012 (14)
C43	0.0782 (18)	0.0707 (17)	0.0793 (18)	0.0019 (15)	0.0096 (15)	−0.0049 (13)
C2	0.0763 (19)	0.093 (2)	0.0745 (18)	0.0062 (16)	0.0033 (15)	−0.0016 (16)
C25	0.080 (2)	0.0744 (19)	0.087 (2)	−0.0147 (15)	−0.0064 (16)	−0.0077 (16)
C1	0.086 (2)	0.121 (3)	0.0722 (18)	−0.0024 (19)	0.0110 (16)	−0.0261 (18)
C24	0.106 (3)	0.107 (3)	0.118 (3)	−0.028 (2)	0.015 (2)	0.000 (2)
N2	0.0712 (13)	0.0541 (11)	0.0558 (11)	0.0035 (10)	0.0176 (10)	0.0101 (9)
N1	0.0462 (10)	0.0529 (11)	0.0588 (11)	0.0045 (9)	0.0130 (9)	0.0007 (9)
N4	0.0630 (12)	0.0545 (12)	0.0589 (12)	0.0016 (10)	−0.0094 (10)	0.0037 (9)
N3	0.0599 (12)	0.0514 (12)	0.0753 (14)	0.0026 (9)	0.0130 (11)	0.0063 (10)
N6	0.0704 (14)	0.0531 (13)	0.0771 (15)	−0.0013 (10)	0.0119 (12)	−0.0038 (11)
N5	0.0544 (13)	0.0635 (14)	0.151 (2)	0.0097 (11)	0.0119 (14)	−0.0127 (15)

### Geometric parameters (Å, º)

**Table:**

C8—C7	1.403 (3)	C45—C46	1.391 (4)
C8—C9	1.405 (3)	C45—H45	0.9300
C8—C17	1.462 (3)	C4—N1	1.454 (3)
C10—C9	1.387 (3)	C4—C3	1.503 (3)
C10—C5	1.418 (3)	C4—H4A	0.9700
C10—C11	1.446 (3)	C4—H4B	0.9700
C11—C16	1.396 (3)	C32—H32	0.9300
C11—C12	1.401 (3)	C22—N3	1.334 (3)
C18—C17	1.323 (3)	C22—H22	0.9300
C18—C19	1.474 (3)	C39—C38	1.387 (4)
C18—H18	0.9300	C39—H39	0.9300
C17—H17	0.9300	C26—C25	1.517 (4)
C12—C13	1.389 (3)	C26—C27	1.517 (4)
C12—N1	1.397 (3)	C26—H26A	0.9700
C16—C15	1.386 (3)	C26—H26B	0.9700
C16—H16	0.9300	C42—C43	1.368 (4)
C15—C14	1.398 (3)	C42—C46	1.402 (4)
C15—N2	1.403 (3)	C42—C41	1.482 (4)
C5—N1	1.377 (3)	C27—N4	1.456 (3)
C5—C6	1.396 (3)	C27—H27A	0.9700
C9—H9	0.9300	C27—H27B	0.9700
C19—C23	1.382 (3)	C41—C40	1.269 (4)
C19—C20	1.388 (3)	C41—H41	0.9300
C7—C6	1.363 (3)	C40—H40	0.9300
C7—H7	0.9300	C3—C2	1.480 (4)
C13—C14	1.380 (3)	C3—H3A	0.9700
C13—H13	0.9300	C3—H3B	0.9700
C20—C21	1.372 (3)	C38—H38	0.9300
C20—H20	0.9300	C29—C30	1.362 (4)
C34—C35	1.386 (3)	C29—H29	0.9300
C34—C37	1.410 (3)	C46—H46	0.9300
C34—C33	1.439 (3)	C30—H30	0.9300
C33—C32	1.396 (3)	C44—N6	1.321 (4)
C33—C28	1.402 (3)	C44—C43	1.350 (4)
C14—H14	0.9300	C44—H44	0.9300
C28—C29	1.388 (3)	C43—H43	0.9300
C28—N4	1.393 (3)	C2—C1	1.534 (4)
C21—N3	1.342 (3)	C2—H2A	0.9700
C21—H21	0.9300	C2—H2B	0.9700
C23—C22	1.369 (3)	C25—C24	1.514 (5)
C23—H23	0.9300	C25—H25A	0.9700
C37—N4	1.378 (3)	C25—H25B	0.9700
C37—C38	1.391 (3)	C1—H1A	0.9600
C35—C36	1.374 (3)	C1—H1B	0.9600
C35—H35	0.9300	C1—H1C	0.9600
C36—C39	1.407 (4)	C24—H24A	0.9600
C36—C40	1.503 (4)	C24—H24B	0.9600
C6—H6	0.9300	C24—H24C	0.9600
C31—C32	1.381 (3)	N2—H2C	0.8600
C31—N5	1.395 (3)	N2—H2D	0.8600
C31—C30	1.402 (4)	N5—H5A	0.8600
C45—N6	1.327 (3)	N5—H5B	0.8600
			
C7—C8—C9	118.4 (2)	N3—C22—H22	117.9
C7—C8—C17	118.7 (2)	C23—C22—H22	117.9
C9—C8—C17	122.8 (2)	C38—C39—C36	123.0 (2)
C9—C10—C5	119.92 (19)	C38—C39—H39	118.5
C9—C10—C11	133.71 (19)	C36—C39—H39	118.5
C5—C10—C11	106.36 (18)	C25—C26—C27	114.7 (2)
C16—C11—C12	119.76 (19)	C25—C26—H26A	108.6
C16—C11—C10	133.52 (19)	C27—C26—H26A	108.6
C12—C11—C10	106.71 (18)	C25—C26—H26B	108.6
C17—C18—C19	126.2 (2)	C27—C26—H26B	108.6
C17—C18—H18	116.9	H26A—C26—H26B	107.6
C19—C18—H18	116.9	C43—C42—C46	116.3 (3)
C18—C17—C8	127.9 (2)	C43—C42—C41	118.7 (3)
C18—C17—H17	116.0	C46—C42—C41	125.1 (3)
C8—C17—H17	116.0	N4—C27—C26	113.6 (2)
C13—C12—N1	129.8 (2)	N4—C27—H27A	108.8
C13—C12—C11	120.9 (2)	C26—C27—H27A	108.8
N1—C12—C11	109.33 (18)	N4—C27—H27B	108.8
C15—C16—C11	119.7 (2)	C26—C27—H27B	108.8
C15—C16—H16	120.1	H27A—C27—H27B	107.7
C11—C16—H16	120.1	C40—C41—C42	124.1 (3)
C16—C15—C14	119.4 (2)	C40—C41—H41	117.9
C16—C15—N2	120.6 (2)	C42—C41—H41	117.9
C14—C15—N2	120.1 (2)	C41—C40—C36	127.2 (3)
N1—C5—C6	130.1 (2)	C41—C40—H40	116.4
N1—C5—C10	109.32 (18)	C36—C40—H40	116.4
C6—C5—C10	120.6 (2)	C2—C3—C4	114.3 (2)
C10—C9—C8	119.7 (2)	C2—C3—H3A	108.7
C10—C9—H9	120.2	C4—C3—H3A	108.7
C8—C9—H9	120.2	C2—C3—H3B	108.7
C23—C19—C20	115.3 (2)	C4—C3—H3B	108.7
C23—C19—C18	120.56 (19)	H3A—C3—H3B	107.6
C20—C19—C18	124.1 (2)	C39—C38—C37	117.3 (2)
C6—C7—C8	123.2 (2)	C39—C38—H38	121.3
C6—C7—H7	118.4	C37—C38—H38	121.3
C8—C7—H7	118.4	C30—C29—C28	118.7 (2)
C14—C13—C12	118.3 (2)	C30—C29—H29	120.7
C14—C13—H13	120.8	C28—C29—H29	120.7
C12—C13—H13	120.8	C45—C46—C42	119.4 (3)
C21—C20—C19	120.1 (2)	C45—C46—H46	120.3
C21—C20—H20	119.9	C42—C46—H46	120.3
C19—C20—H20	119.9	C29—C30—C31	122.4 (2)
C35—C34—C37	120.0 (2)	C29—C30—H30	118.8
C35—C34—C33	133.2 (2)	C31—C30—H30	118.8
C37—C34—C33	106.7 (2)	N6—C44—C43	125.6 (3)
C32—C33—C28	120.0 (2)	N6—C44—H44	117.2
C32—C33—C34	133.5 (2)	C43—C44—H44	117.2
C28—C33—C34	106.4 (2)	C44—C43—C42	119.8 (3)
C13—C14—C15	121.9 (2)	C44—C43—H43	120.1
C13—C14—H14	119.0	C42—C43—H43	120.1
C15—C14—H14	119.0	C3—C2—C1	112.9 (3)
C29—C28—N4	130.1 (2)	C3—C2—H2A	109.0
C29—C28—C33	120.4 (2)	C1—C2—H2A	109.0
N4—C28—C33	109.5 (2)	C3—C2—H2B	109.0
N3—C21—C20	124.6 (2)	C1—C2—H2B	109.0
N3—C21—H21	117.7	H2A—C2—H2B	107.8
C20—C21—H21	117.7	C24—C25—C26	114.2 (3)
C22—C23—C19	121.0 (2)	C24—C25—H25A	108.7
C22—C23—H23	119.5	C26—C25—H25A	108.7
C19—C23—H23	119.5	C24—C25—H25B	108.7
N4—C37—C38	129.9 (2)	C26—C25—H25B	108.7
N4—C37—C34	109.4 (2)	H25A—C25—H25B	107.6
C38—C37—C34	120.7 (2)	C2—C1—H1A	109.5
C36—C35—C34	120.6 (2)	C2—C1—H1B	109.5
C36—C35—H35	119.7	H1A—C1—H1B	109.5
C34—C35—H35	119.7	C2—C1—H1C	109.5
C35—C36—C39	118.2 (2)	H1A—C1—H1C	109.5
C35—C36—C40	117.4 (2)	H1B—C1—H1C	109.5
C39—C36—C40	124.3 (2)	C25—C24—H24A	109.5
C7—C6—C5	118.1 (2)	C25—C24—H24B	109.5
C7—C6—H6	120.9	H24A—C24—H24B	109.5
C5—C6—H6	120.9	C25—C24—H24C	109.5
C32—C31—N5	121.5 (2)	H24A—C24—H24C	109.5
C32—C31—C30	119.0 (2)	H24B—C24—H24C	109.5
N5—C31—C30	119.5 (2)	C15—N2—H2C	120.0
N6—C45—C46	122.9 (3)	C15—N2—H2D	120.0
N6—C45—H45	118.5	H2C—N2—H2D	120.0
C46—C45—H45	118.5	C5—N1—C12	108.27 (17)
N1—C4—C3	114.8 (2)	C5—N1—C4	127.33 (19)
N1—C4—H4A	108.6	C12—N1—C4	124.32 (19)
C3—C4—H4A	108.6	C37—N4—C28	107.96 (19)
N1—C4—H4B	108.6	C37—N4—C27	126.1 (2)
C3—C4—H4B	108.6	C28—N4—C27	125.9 (2)
H4A—C4—H4B	107.5	C22—N3—C21	114.8 (2)
C31—C32—C33	119.6 (2)	C44—N6—C45	115.9 (2)
C31—C32—H32	120.2	C31—N5—H5A	120.0
C33—C32—H32	120.2	C31—N5—H5B	120.0
N3—C22—C23	124.2 (2)	H5A—N5—H5B	120.0
			
C9—C10—C11—C16	−3.2 (4)	C10—C5—C6—C7	−1.7 (3)
C5—C10—C11—C16	178.0 (2)	N5—C31—C32—C33	179.7 (2)
C9—C10—C11—C12	178.4 (2)	C30—C31—C32—C33	−1.6 (3)
C5—C10—C11—C12	−0.4 (2)	C28—C33—C32—C31	0.5 (3)
C19—C18—C17—C8	−178.2 (2)	C34—C33—C32—C31	177.2 (2)
C7—C8—C17—C18	163.1 (2)	C19—C23—C22—N3	−0.4 (4)
C9—C8—C17—C18	−14.6 (4)	C35—C36—C39—C38	2.3 (4)
C16—C11—C12—C13	0.4 (3)	C40—C36—C39—C38	−173.6 (2)
C10—C11—C12—C13	179.04 (19)	C25—C26—C27—N4	61.0 (3)
C16—C11—C12—N1	−178.96 (18)	C43—C42—C41—C40	−160.0 (3)
C10—C11—C12—N1	−0.3 (2)	C46—C42—C41—C40	19.3 (4)
C12—C11—C16—C15	−0.2 (3)	C42—C41—C40—C36	174.1 (2)
C10—C11—C16—C15	−178.4 (2)	C35—C36—C40—C41	−151.0 (3)
C11—C16—C15—C14	−0.3 (3)	C39—C36—C40—C41	24.9 (4)
C11—C16—C15—N2	−178.79 (19)	N1—C4—C3—C2	−63.4 (3)
C9—C10—C5—N1	−177.99 (18)	C36—C39—C38—C37	1.4 (4)
C11—C10—C5—N1	1.0 (2)	N4—C37—C38—C39	174.3 (2)
C9—C10—C5—C6	2.5 (3)	C34—C37—C38—C39	−3.2 (3)
C11—C10—C5—C6	−178.5 (2)	N4—C28—C29—C30	−178.0 (2)
C5—C10—C9—C8	−1.2 (3)	C33—C28—C29—C30	−0.2 (4)
C11—C10—C9—C8	−179.9 (2)	N6—C45—C46—C42	0.8 (4)
C7—C8—C9—C10	−0.8 (3)	C43—C42—C46—C45	−1.4 (4)
C17—C8—C9—C10	176.8 (2)	C41—C42—C46—C45	179.3 (2)
C17—C18—C19—C23	176.3 (2)	C28—C29—C30—C31	−0.9 (4)
C17—C18—C19—C20	−3.5 (4)	C32—C31—C30—C29	1.8 (4)
C9—C8—C7—C6	1.7 (3)	N5—C31—C30—C29	−179.4 (3)
C17—C8—C7—C6	−176.0 (2)	N6—C44—C43—C42	0.1 (4)
N1—C12—C13—C14	179.1 (2)	C46—C42—C43—C44	0.9 (4)
C11—C12—C13—C14	−0.1 (3)	C41—C42—C43—C44	−179.7 (3)
C23—C19—C20—C21	0.9 (3)	C4—C3—C2—C1	−171.7 (2)
C18—C19—C20—C21	−179.3 (2)	C27—C26—C25—C24	173.8 (3)
C35—C34—C33—C32	−1.8 (4)	C6—C5—N1—C12	178.2 (2)
C37—C34—C33—C32	−177.7 (2)	C10—C5—N1—C12	−1.2 (2)
C35—C34—C33—C28	175.2 (2)	C6—C5—N1—C4	−5.0 (4)
C37—C34—C33—C28	−0.7 (2)	C10—C5—N1—C4	175.5 (2)
C12—C13—C14—C15	−0.3 (3)	C13—C12—N1—C5	−178.3 (2)
C16—C15—C14—C13	0.5 (3)	C11—C12—N1—C5	0.9 (2)
N2—C15—C14—C13	179.1 (2)	C13—C12—N1—C4	4.8 (4)
C32—C33—C28—C29	0.4 (3)	C11—C12—N1—C4	−175.9 (2)
C34—C33—C28—C29	−177.1 (2)	C3—C4—N1—C5	103.0 (3)
C32—C33—C28—N4	178.61 (19)	C3—C4—N1—C12	−80.8 (3)
C34—C33—C28—N4	1.2 (2)	C38—C37—N4—C28	−176.9 (2)
C19—C20—C21—N3	0.3 (4)	C34—C37—N4—C28	0.7 (2)
C20—C19—C23—C22	−0.9 (3)	C38—C37—N4—C27	4.4 (4)
C18—C19—C23—C22	179.3 (2)	C34—C37—N4—C27	−178.0 (2)
C35—C34—C37—N4	−176.53 (19)	C29—C28—N4—C37	176.8 (2)
C33—C34—C37—N4	0.0 (2)	C33—C28—N4—C37	−1.2 (2)
C35—C34—C37—C38	1.4 (3)	C29—C28—N4—C27	−4.5 (4)
C33—C34—C37—C38	177.9 (2)	C33—C28—N4—C27	177.5 (2)
C37—C34—C35—C36	2.4 (3)	C26—C27—N4—C37	84.9 (3)
C33—C34—C35—C36	−173.0 (2)	C26—C27—N4—C28	−93.6 (3)
C34—C35—C36—C39	−4.2 (3)	C23—C22—N3—C21	1.6 (4)
C34—C35—C36—C40	172.0 (2)	C20—C21—N3—C22	−1.5 (4)
C8—C7—C6—C5	−0.5 (4)	C43—C44—N6—C45	−0.7 (4)
N1—C5—C6—C7	179.0 (2)	C46—C45—N6—C44	0.2 (4)

### Hydrogen-bond geometry (Å, º)

**Table:**

*D*—H···*A*	*D*—H	H···*A*	*D*···*A*	*D*—H···*A*
N2—H2*D*···N6	0.86	2.56	3.154 (3)	128
N5—H5*B*···N3^i^	0.86	2.36	3.163 (3)	156

Symmetry code: (i) −*x*+1, *y*+3/2, −*z*+1/2.
