# Supplementary figures and images for: Crystal structure of 9-butyl-6-[2-(pyridin-4-yl)ethen­yl]carbazol-3-amine
Source: Acta Crystallogr E Crystallogr Commun. 2015 Apr 25;71(Pt 5):o345–6. doi: 10.1107/S2056989015007975 (PMC4420045; doi:10.1107/S2056989015007975)

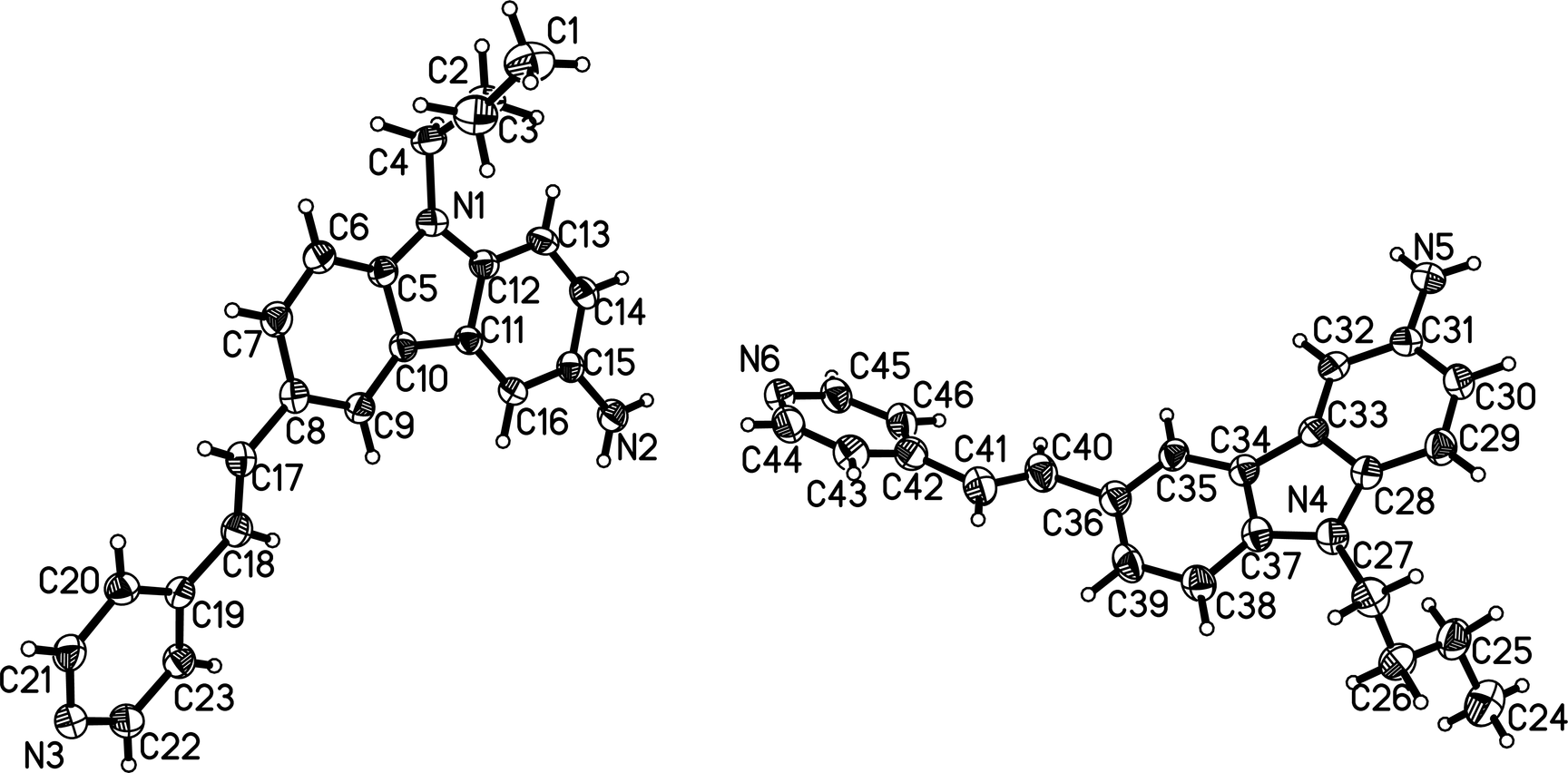

Supplement: Supplementary file 4 [file e-71-0o345-fig1.tif]
